# Sarcopenia Incidence From Age 65 to 90+ Years From 2011 to 2023 in the ARIC Cohort

**DOI:** 10.1093/geroni/igaf122.1219

**Published:** 2025-12-31

**Authors:** Jordan Weiss, Shoshana Ballew, Hubert Leo, Morgan Grams, B Gwen Windham, Lynne Wagenknecht, Josef Coresh, Marcus Goncalves

**Affiliations:** NYU Grossman School of Medicine, New York, New York, United States; New York University, New York, New York, United States; New York University, New York, New York, United States; New York University, New York, New York, United States; University of Mississippi Medical Center - The MIND Center, Jackson, Mississippi, United States; Wake Forest University School of Medicine, Winston-Salem, North Carolina, United States; New York University, New York, New York, United States; New York University, New York, New York, United States

## Abstract

Sarcopenia, characterized by progressive loss of skeletal muscle mass and function, is a major contributor to disability and mortality in aging populations. Incidence rates at the oldest ages from recent studies are limited and needed to identify at-risk populations and inform interventions and policy. We estimated age-specific sarcopenia incidence by race and sex in the Atherosclerosis Risk in Communities (ARIC) study. Sarcopenia was defined using Sarcopenia Definitions and Outcomes Consortium (SDOC) criteria: low grip strength (<35.5 kg for males, <20 kg for females) and slow gait speed (<0.8 m/s). Among 5,985 adults (mean age, 75.4 [SD, 5.1] years; 57.9% female; 21.9% Black) at baseline (visit 5, 2011–2013), 665 (11.1%) had prevalent sarcopenia with prevalence rising from 5.0% at age 65-69 to 36.0%at age 85-89 years. Incident cases were identified through visit 10 (2022–2023). Over 30,727 person-years (PY), 900 incident cases were identified (incidence rate [IR], 2.92 per 100 PY; 95% CI, 2.74–3.13). Incidence increased with age. Among participants aged 60–69 at baseline, IRs were highest in Black males (2.57; 95% CI, 1.55–4.27) and lowest in Black females (1.13; 95% CI, 0.64–1.99). By ages 80–89, all rates rose to approximately 5% a year except for Black females (2.87; 95% CI, 1.73–4.76). Targeted prevention and early interventions are needed to mitigate disparities and reduce sarcopenia-related disability in aging populations.

